# Economic analysis on the causes of mental health stress of enterprise employees based on emotional feature clustering

**DOI:** 10.3389/fpsyg.2022.990203

**Published:** 2022-09-23

**Authors:** Benqing Li, Yajie Qiao

**Affiliations:** ^1^School of Economics and Management, Northwest University, Xi'an, China; ^2^School of Economics and Management, Ankang University, Ankang, China

**Keywords:** emotional clustering, enterprise employees, causes of psychological stress, economics, mental health

## Abstract

Emotional labor generally exists in organization members. Emotional labor will not only affect employees’ interpersonal relationships, but also affect employees’ mental health. Affected by many factors such as the economic environment, they often need to bear multiple pressures. The degree of stress is positively correlated with the depth of the development of the times and people’s education. As mental health research has become the frontier and hot spot in the field of psychology, the role of mental health in the process of employee creativity has been paid more and more attention. Therefore, this paper proposes a study on the causes of employees’ psychological stress based on emotional feature clustering. Based on the clustering of emotional characteristics, this paper analyzes the causes of employees’ mental health stress from an economic perspective. For teams with low level of team openness, with the improvement of team heterogeneity, team task performance shows a slight upward trend. It is clear from the experiment that when the number of experiments reaches 100, the task performance of high atmosphere level is 12.14, while the task performance of low atmosphere level is only 9.89. Therefore, the atmosphere of team employees is very important to team task performance. Through the cluster analysis of employees’ mental health characteristics, it not only increases employees’ spare time life, but also reduces employees’ daily contradictions. It eases the work pressure of employees, and becomes a platform for employees to improve their awareness and a promoter of harmonious employee relations.

## Introduction

As China continues to deepen the development of the socialist market economy and promote the process of reform and opening up, the organizational form of Chinese enterprises is quietly undergoing profound changes, becoming more and more complex, and becoming a situation faced by various relationships within enterprises. In the new era, enterprise employees also need to face many problems they have never encountered. Enterprise employees cannot live without the complex social environment. Affected by many factors such as the economic environment, they often need to bear multiple mental health pressures (Kim et al., 2022). The degree of stress is positively correlated with the depth of the development of the times and people’s education. In the era of extreme material shortage, few people talk about pressure; Even in the era of fierce material competition, the people who talk about the most pressure are not those who lack material, but those who do not lack material and spiritual life, that is, those who often say white-collar and senior intellectuals (Qin et al., 2018; Yeh et al., 2020). Employees’ low mental health level, poor sense of responsibility and low emotional management ability are the biggest hidden dangers of enterprise safety. Responsibility and emotional management ability belong to the category of personality and mental health. Employees with sound personality and mental health have good psychological flexibility and emotional management ability, which can effectively reduce the occurrence of safety accidents outside the production field. The various troubles of enterprise employees are also caused by the superposition of various pressures, which damage the mental health of enterprise employees and have a negative impact on their work attitude (Yao et al., 2019). In the practice of stress management, organizations and individuals are targeted, and many enterprises have taken and implemented various measures. However, enterprise managers are still troubled by employee pressure, and some measures only lasted a short time to solve employee problems.

Researchers have always believed that positive emotions can promote employees’ creative activities. Individuals in a positive emotional state will show more creativity in a series of tasks, while those in other emotional states will not. This view is supported by many empirical studies, for example, if subjects are required to generate more ideas. Instead of completing practical creative products, positive emotions will trigger more creative activities. In addition, the study also found that positive emotions can help solve innovation problems. The reason why positive emotions can promote creative activities is that they can make individuals’ thoughts more divergent, recall more and more extensive information, and make new connections between seemingly unrelated information. Therefore, individuals can identify problems more effectively, integrate various resources, and generate higher creativity. As emotion research has become the frontier and hot spot in the field of psychology, the role of emotion in the process of employee creativity has attracted more and more attention. Therefore, this paper proposes to study the causes of employees’ psychological stress based on emotional feature clustering. In today’s rapidly changing environment, creativity is not only a necessary condition for organizations to win competitive advantage, but also the key for organizations to survive. As a psychological phenomenon, creativity is influenced by many factors, such as intelligence, cognition, cognitive style, personality, motivation, emotion, environment and so on (Lorber et al., 2018; Shu, 2021). When clustering the emotional characteristics of employees’ psychological stress, we need to know what kind of relationship exists between emotional characteristics and employees’ psychological stress. Therefore, we believe that the tendency of employees’ psychological stress where the emotional characteristics are located also plays a certain role in the emotional tendency of the characteristics. Enterprise employees must face faster and faster work rhythm and bear greater and greater pressure. The work becomes monotonous and boring, and is prone to physical, mental and emotional problems. When employees have related symptoms, we can judge that employees are under great pressure to lose vitality. Therefore, it has become an important aspect of enterprise human resource management to pay attention to employee stress management. Excessive and continuous stress can lead to serious physical and mental illness of employees, and stress and emotion management can prevent the destructive damage caused by stress to employees. The co-occurrence matrix is constructed according to the co-occurrence relationship of affective features, and then the extended features for classification are generated by affective feature clustering method. The emotion analysis method based on emotion dictionary is used to analyze the user’s emotion, and the positive emotion score and negative emotion score are calculated for each user’s reply. Calculate the number of positive emotion words and negative emotion words, and finally calculate the average value with the user as the unit. Users can find information, share views, evaluate and discuss hot topics on the platform. These behaviors will generate a large amount of text data containing user emotions. By analyzing these data, we can understand the emotional behavior characteristics of users and grasp the views and emotional tendencies of user groups on hot topics. Get the emotional feature vector of each user. Finally, according to the emotional feature vector of users, clustering analysis is carried out on users. Combined with the classification features in the original training domain, a new affective classifier is trained; The classification features of the original training domain form another classifier; The classifiers work together to complete the final emotion classification (Рогач et al., 2018). In the existing research on emotion and creativity, the relationship between emotion with different potency and employee creative performance is the main focus of disagreement in the existing research results. Therefore, based on the clustering of emotional characteristics, this paper analyzes the economic causes of employees’ psychological stress.

The mental health stress of enterprise employees is understood as the physiological and psychological response of individuals to external stimuli, which is the result of stressor stimulation; According to the theory of subjective characteristics, stress is related to some characteristics of individuals and is generated by the interaction between specific environments and specific people. When a person evaluates the environment, the internal and external environmental needs exceed his resources and abilities, which will produce psychological pressure (Jilan and Wen, 2018). Generally speaking, under the condition of a certain technical level, only the input of wages continues to increase, while the input of other factors remains unchanged. Then, when the wage increases to a certain extent, if the wage is increased again, the incentive effect caused by the wage increase will gradually decrease until a negative number (Liu et al., 2017; Tabianan et al., 2022). There have been many studies on the relationship between emotion and employees’ creative compensation. The difference mainly focuses on whether positive emotions or negative emotions promote creativity. Under this difference, enterprise managers do not know how to stimulate effective emotional content in management, so as to improve the level of creative compensation of the organization. By clustering the economic emotional characteristics of employees’ psychological stress, the number of clusters that can be obtained is limited. Then, if the economic emotional characteristics of employees’ psychological stress appear in a cluster, the problem of sparse features can be alleviated by expressing binary values as features and extending them to the original training feature set.

The innovation of this paper is to propose the relationship between characteristic emotional mental health factors and information related performance. Through the emotional characteristics of each voice, we can build the overall emotional characteristics of users. The overall emotional characteristics of users help us analyze users’ emotions. This paper analyzes the reasons why employees’ rights and interests are easy to be damaged. As the principal, the enterprise cannot directly interfere with the agent’s behavior, and can only encourage the agent to choose the behavior that can achieve the principal’s goal through an appropriate salary structure. The analysis of principal-agent principle shows that any kind of institutional arrangement and policy measures are necessary in the construction of market economy. Only when the participation constraints and incentive compatibility are satisfied, it is possible to effectively motivate people to work hard. We have formulated a good incentive mechanism, guarantee mechanism, management mechanism and development mechanism to ensure the good independent operation of the market economy. The research explains the characteristics of the labor market of private enterprises, and provides a reference value for analyzing the imbalance between supply and demand in the labor market.

### The overall structure of this paper consists of five parts

The first chapter introduces the background and significance of employees’ mental health stress, and then introduces the main work of this paper. The second chapter focuses on the related work of psychological stress of enterprise employees. The third chapter introduces the method proposed in this paper, constructs a psychological stress model with characteristic emotional tendency, and analyzes the reasons why the rights and interests of enterprise employees are easy to be damaged. The fourth chapter studies the economics of the causes of employees’ psychological stress, carries out simulation experiments and discusses the results. The fifth chapter is a summary of the full text.

## Related work

At present, the research on the causes of employees’ psychological stress mainly focuses on the emotional analysis of employees, and most of them only take it as a dimension of organizational commitment. The research on emotional analysis of employees’ psychological stress has just started, mainly drawing lessons from previous research methods and directions on employees’ psychological stress. Here are some previous research contents.

Adebayo et al. pointed out that the stressors of employees in enterprises come from their work and life, and they have different intensity characteristics. The pressure source belongs to the type of force majeure crisis, which makes the increase of employees’ pressure depend on two ways: First, the force majeure crisis causes losses to employees’ or relatives’ property, or personal injury, which directly affects employees’ working and living environment, and also affects employees’ spirit (Adebayo et al., 2019). Kim et al., thinks that personal characteristics, internal and external organizational factors are the three major factors that cause occupational stress, among which internal organizational factors are the direct cause of occupational stress (Sharma and Sharma, 2020). Ting et al. research on the relationship between job stress and peripheral performance and the introduction of emotional intelligence as moderating variable can further enrich and improve the theoretical research results of job stress and job performance and expand the scope of its moderating variables (Ting et al., 2019). Budimir et al., believes that in the past 10 years, employee service projects specializing in complex work have emerged, and these services have been recognized by trade unions, public departments and private individuals. These programs are not unique, and they cannot solve the recently discovered problems such as alcoholism and psychological problems. At present, the solution to these problems is the embodiment of unique aspects (Budimir et al., 2018). Silva et al. shows that employees’ job stress can be divided into six aspects, such as job stress, interpersonal stress and career development stress, which provides enterprises with the breakthrough point of employee job stress management from different angles (Silva et al., 2018). Ling et al. thinks that employees’ “commitment” to the organization is more manifested as employees’ emotional dependence on the organization. Employees are reluctant to leave the organization, not because they have invested too much in the organization unilaterally, or because they are worried about losing benefits such as pensions, but mainly because employees have a lot of emotional dependence on the organization (Ling et al., 2017). Saha et al. pointed out that moral and practical problems have become the focus of social work services, but the specific solutions to these problems have not been proved by empirical research. Although personal stress is closely related to employee stress, the intervention of employee assistance program has played a role in improving employee productivity, but it has not played a role in reducing employee stress (Saha et al., 2021). Wang et al. put forward the inverted theory of job stress and job performance. Only moderate job stress will produce higher performance, while insufficient or excessive job stress will have adverse effects on employees’ performance level (Wang et al., 2021). Durdyev et al. Different employees have different forms of commitment, and at the same time, the reasons for different commitments are also different. In an empirical study, they analyzed dozens of influencing factors, and found that the factors influencing emotional commitment mainly include the challenge of work, the clarity of position, the clarity of goal, etc. (Durdyev and Ismail, 2019). Adiguzel and Küükolu put forward that the “commitment” of enterprise employees’ psychological pressure is named as “continuous commitment,” that is, a commitment that employees have to stay in the organization in order to maintain their position in the organization and the welfare benefits gained from years of investment. Psychological pressure is a commitment based on material interests and with strong trading color (Adiguzel and Küükolu, 2020).

In view of the previous studies, this paper studies the causes of employees’ psychological stress from the angle of economics based on emotional feature clustering. From the individual point of view, employees’ poor psychological quality and low bearing capacity are an important factor affecting employees’ psychology. Before the analysis of stress, make assumptions about its general process, and give an example to illustrate the process of a manager’s stress. The enterprise manager is engaged in management and decision-making, and provides him with a relatively generous reward, the basic salary of which is higher than that of ordinary employees. The main task given to him is to decide whether to invest in a certain project, including organizational management. Generally, employees’ income is relatively low. Under other similar conditions, the low basic income makes them pay more attention to income, that is, the diminishing marginal utility is not as fast as that of managers, and it is reflected in the utility curve that the radian is not as big as that of managers. Different methods should be adopted for different psychological states, such as stressing the advantages of employees who are worried too much, helping them to go all out, stressing the disadvantages of some blindly optimistic employees, helping them to make up for the shortcomings in their work and life, striving for greater progress, neither avoiding problems nor exaggerating them, so that employees can let go of their ideological burdens, fully realize their own problems and harms, and help them establish a correct world outlook, outlook on life and values, and the ability to identify unhealthy social phenomena.

## Materials and methods

The clustering of emotional characteristics needs to understand the causes of employees’ mental health stress, but this relationship is more complex, and it is not comprehensive to describe it only by emotional characteristics. Enterprise employees must face faster and faster work pace and bear more and more pressure. Work becomes monotonous and boring, and it is easy to show physical, mental and emotional problems. When employees show relevant symptoms, it can be judged that employees are under great pressure to lose vitality. Therefore, paying attention to employee stress management has become an important aspect of enterprise human resource management. Excessive and continuous stress will lead to serious physical and mental illness of employees, and stress and emotional management can prevent the devastating damage caused by stress to employees. The evaluation object, evaluation characteristics, opinion words and other words used in the evaluation are closely related to the domain knowledge of the product. Considering the role of mental health analysis in the causes of psychological stress of enterprise employees, this paper takes the clustering characteristics of emotional characteristics as eigenvalues for analysis.

### Psychological stress model based on emotional tendency expression of characteristic mental health

In this paper, a local feature word selection method based on random forest, mutual information and co word analysis is proposed for text classification based on digital document data. This method not only considers the correlation between keywords and document categories, but also considers the co-occurrence degree between different keywords. Combined with these two points, the final keyword subset is used as the feature variable of vector space. It can not only effectively solve the high-dimensional problem of text representation, but also reduce the noise information in the data, so as to mine more classification information. The local feature word selection method proposed in this paper is applied to text classification. It can not only reduce the computational cost of training the classifier, but also improve the prediction accuracy of the samples to be classified. The evaluation words are extracted by the method of restricting the left and right windows of the feature words. In this paper, four words are taken from the left and right windows of the feature words. The experiment found that the evaluation words appeared in front of the feature words were relatively few, and the extracted relevance relationship was often not the collocation relationship in the actual sense, so we only considered the evaluation words in the back. Next, it is obvious that there are both positive and negative in this sentence, and a score cannot be used to express its emotional tendency (Norouzianpour, 2019). And the setting of this weight will also affect the final emotional score. The sensitivity is too high. Therefore, the final correct treatment of this sentence is to get a positive score and a negative score of this sentence, so the negative score is also a positive number, so there is no need to use a negative number. They also represent the emotional tendency of this sentence. The emotional tendency of a feature is not only related to the emotional tendency of the feature itself, but also related to the sentence and context in which the feature is located. When we calculate the eigenvalues, we should comprehensively express the emotional tendency of the features.

On the one hand, the result variables of diversity impact are divided into dimensions according to the criteria related to information utilization, on the other hand, the research levels are divided according to the different roles of individuals and teams. Therefore, information related performance is operationally defined as employee learning performance and individual innovation behavior at the individual level, and team task performance at the individual level, So as to form a transmission mechanism between different levels of diversity related performance (Xing et al., 2018). Words or phrases with the same emotional tendency are more likely to appear in the same product reviews. For example, in general, the probability of “very good” and “perfect” appearing in the same reviews with positive emotional tendency is greater than the probability of “very poor” and “perfect” appearing in the same review. Self-function adjustment becomes the driving force of personality development. The self-regulation system of personality is the internal factor of personality development. Personality control system is centered on self-awareness. Self-consciousness is the consciousness of people about themselves and the relationship between themselves and the objective world. It has three subsystems: self-awareness, self-experience and self-control. Guide employees to maintain their mentality at a healthy and mature level, so that they can understand and face problems, give full play to their potential, and make better use of social resources and opportunities in problem solving, so as to improve their self-confidence and quality of life to a higher level (Adiguzel and Kucukoglu, 2020).

**Figure 1:**
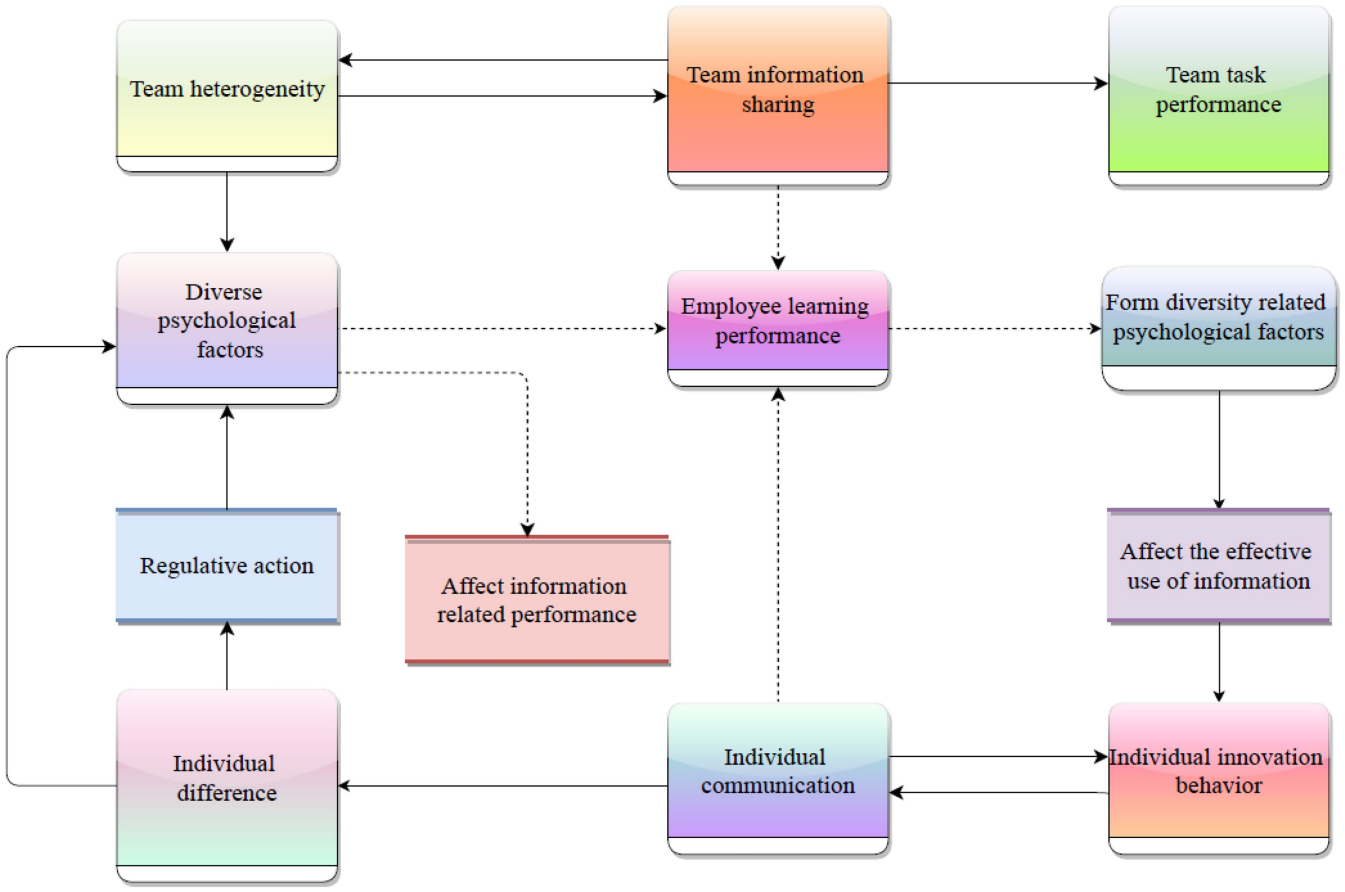
Based on the conceptual model of the relationship between characteristic emotional psychological factors and information-related performance.

The emotional scores of comments are added by different clauses. Therefore, in order to obtain the emotional score of the comment, we must first calculate the emotional score of each sentence in the comment. There are also some emotional features in the negative context, that is, there are some negative deictics. To analyze the emotional tendency of comments, we must first match the emotional words. The method of dictionary matching is mainly used. The emotional vocabulary used in this case is the word set for emotional analysis. It mainly uses the vocabulary of “Chinese positive evaluation,” “Chinese negative evaluation,” “Chinese positive emotion,” “Chinese negative emotion,” etc. Combine the two words lists of “Chinese positive evaluation” and “Chinese positive emotion,” and give each word an initial weight of 1 as the positive comment emotion word list of this case. Merge the two words lists of “Chinese negative evaluation” and “Chinese negative emotion,” and give each word an initial weight of −1 as the negative comment emotion word list of this case. These adjectives and verbs together constitute emotional features. The social classification process caused by the diversity of employees may have a negative impact on the effective use of information. Whether this negative impact will significantly affect the potential advantages of diversity will be affected by deep-seated mental health factors, including the attitude of team members towards diversity. Therefore, the psychological dimension related to the diversity of team members will play a regulatory role in the relationship between diversity and effective use of information (Rana and Singh, 2021). Based on the above analysis, this paper constructs a conceptual model of the effective use of information in the employee diversification effect, as well as the relationship between diversification related mental health factors and performance, as shown in Figure 1.

Through the emotional characteristics of each speech, we can construct the overall emotional characteristics of users, and calculate the positive emotional mean, negative emotional mean, positive emotional variance, negative emotional variance, average positive emotional words and negative emotional words, respectively. The overall emotional characteristics of users are helpful for us to analyze the emotions of users. If the accuracy can be regarded as a continuous variable, and the variable corresponding to the accuracy is p, then the expected E(X) of X=pq is


(1) $$E\left(X\right)=\overset{1}{\underset{a}{\int }}pq\rho \left(p,q\right)$$

The relevant performance expectation E(Y) of the same Y=(1−p)(1−q) is


(2) $$E\left(Y\right)=\overset{1}{\underset{a}{\int }}\left(1-p\right)\left(1-q\right)=\frac{\left(1-a^{2}\right)}{2}$$

If the accuracy is greater than 1/2, that is, when 1/2 < *p* < 1, otherwise ρ(x,y)=0, then


(3) $$E\left(X\right)=\overset{1}{\underset{\frac{1}{2}}{\int }}pq\rho \left(p,q\right)=9/64$$

Similarly, the relevant performance expectations of Y=(1−p)(1−q) are


(4) $$E\left(Y\right)=\overset{1}{\underset{\frac{1}{2}}{\int }}\left(1-p\right)\left(1-q\right)=1/64$$

At this time, the ratio of E(X) to E(Y) can be obtained as follows


(5) $$T=\frac{E\left(X\right)}{E\left(Y\right)}=9$$

The average scores of these emotional features are obtained from the initial training set.


(6) $$f_{i}=\frac{\underset{1\leq i\leq R_{i}}{\sum }}{\left|R_{i}\right|}$$

The average score of $f_{i}$ here, $R_{i}$ represents the set of comments containing feature $f_{i}$, and $r_{i}$ represents the score of comments containing feature $f_{i}$.

Integrate employee diversity, effective use of information, diversity psychological factors and information related performance, and focus on the path mechanism of cross-level interaction of variables on the basis of studying the relationship between variables, so as to open the “black box” of diversity effect and solve the “double-edged sword effect” of employee diversity. If the average number of negative affective words or the average number of positive affective words is very high, but the average number of positive affective words and negative affective words are not high, it means that many negative words are used in the expression of affective words, which means that they are euphemistic (Ferry et al., 2019). On the contrary, if the mean value of positive emotion or negative emotion is high, and the mean number of positive emotion words and negative emotion words are also high, it means that it is more direct in expressing emotion.

Affective words with similar affective tendencies have a higher probability of appearing in the same affective tendencies; Then these “similar” emotional features can be clustered by their co-occurrence in the comments. The purpose of corporate social work is to help employees’ psychological stress and information related performance and resources to solve problems that cannot be solved. In the process of assistance, the professional relationship between case workers and case owners has the characteristics of face-to-face or one-to-one. A working relationship established between social workers and the case owner. Through the dynamic interaction between the inner feelings and emotions of both sides, it helps the case owner to solve problems and improve his personal ability. Relying on the use of psychological professional knowledge and methods, we can help to improve the environment for the psychological causes of maladjusted employees, Promote the further improvement of employees’ psychological stress and information related performance (Cetin, 2018).

### An analysis of the reasons why the rights and interests of enterprise employees are easy to be damaged

**Figure 2:**
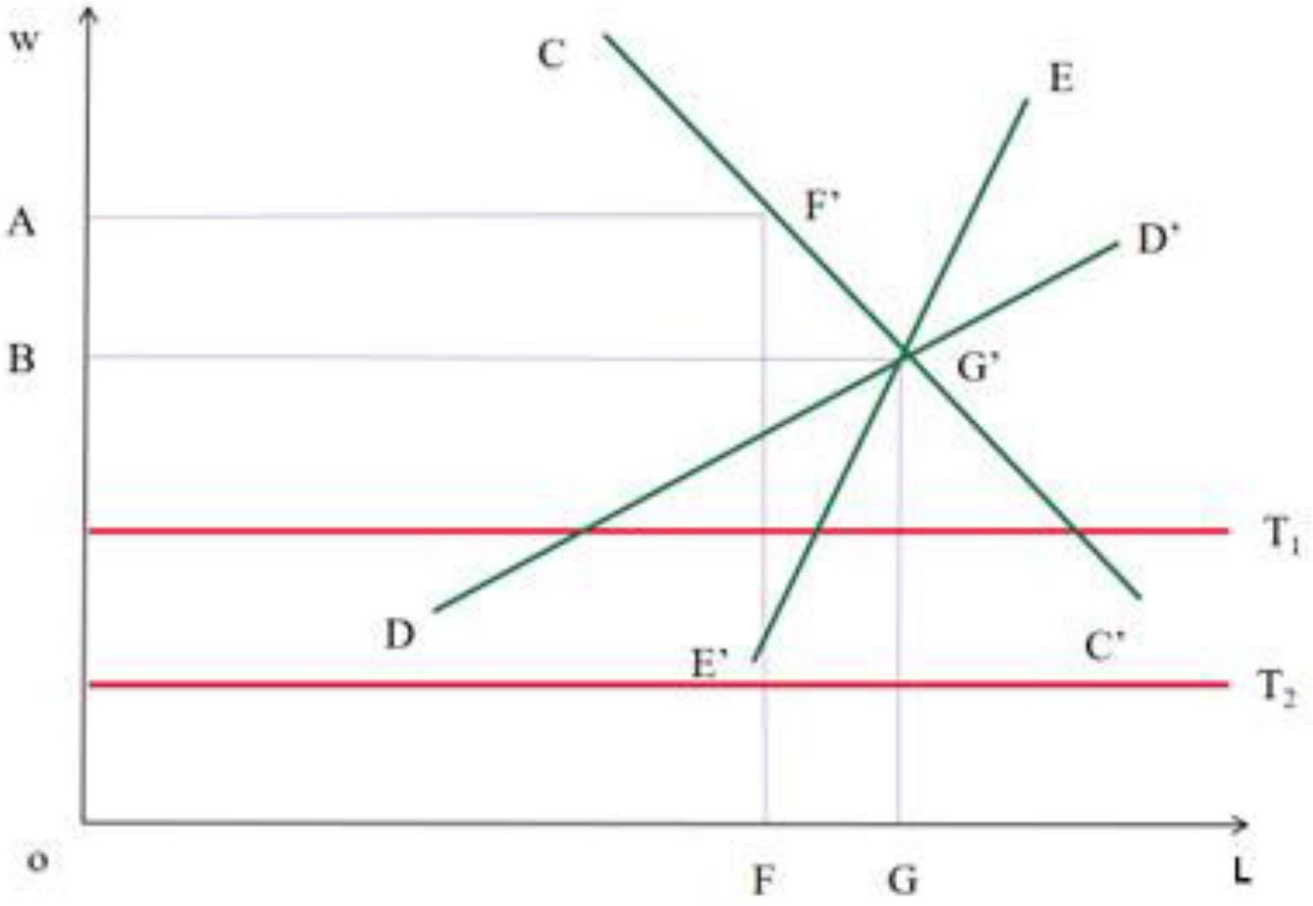
Labor market of enterprise employees.

The general root of the infringement of the rights and interests of employees in private enterprises lies in the long-term absence of various systems. Including labor contract system, wage system, security system, etc. Since the implementation of the market economy system in China, the arrangement of the economic system has obviously lagged behind the rapid economic development. The original labor management system can no longer adapt to the new labor relations. However, the implementation of the labor contract law is a good remedy. In the new era, enterprise social work belongs to the category of enterprise management innovation. It is an emerging force to deal with enterprise contradictions. It takes the introduction of professional social work concepts and methods as a platform to manage employees’ adaptation to production. Its purpose is to improve enterprise efficiency on the premise of developing employees’ career and welfare. This is just consistent with the problems faced by enterprises in the new era. In terms of enterprise emergency response, The status and role of social workers cannot be ignored and play an irreplaceable role (Khalid et al., 2020; Christensen et al., 2021). Firstly, it is assumed that both the labor market and the product market are perfectly competitive markets. Secondly, in order to explain the characteristics of the labor market of private enterprises, and then analyze the imbalance between supply and demand in the labor market. A simple labor supply and demand model is introduced, as shown in Figure 2.

The horizontal axis represents the labor supply quantity L, and the vertical axis represents the wage level W of the labor force in the labor market. CC’ line and EE’ are the supply curve of labor force, but also the cost line and wage line of labor force, DD’ line is the demand curve of labor force, and its intersection point G’ is the equilibrium point of labor force supply and demand, OG is the number of employees employed, OB represents the wage level of employees on this scale, so the area OCGG; It is the total wage cost of employees paid by the business owner. The minimum living security line of employees can be expressed by *T*_1_ and *T*_2_, and its meaning needs to meet two conditions: First, in order to avoid that the supply of labor force is not driven by interests, it must not be lower than or equal to the wage level of industries or departments with the lowest social wage rate. Investigating the present situation of the enterprise labor market, it is not difficult to find that the enterprise labor market has the following two obvious characteristics.

1. In the competitive labor market under the condition of market economy, the motivation of employers to reduce labor costs always exists, and both employers and employees are independent rational economic men; Compared with state-owned enterprises, private enterprises are economic entities with clear property rights, self-financing and self-risk.
2. Poor market development and lack of legal protection have created relaxed employment conditions for enterprise owners. Because employees are unprotected and their economic strength is relatively low, it is conceivable that their ability to defend their rights is very low when faced with employers with strong industries. Obviously, there is no trade union or similar organization that can fully represent the interests of employees and have the strength to negotiate and compete with business owners in the market.

As the principal of an enterprise, it cannot directly interfere with the agent’s behavior, but can only encourage the agent to choose the behavior that can achieve the principal’s goal through the appropriate reward structure. However, the agent’s behavior goal is to maximize personal utility. Therefore, if the first derivative of the agent’s expected utility is zero, the incentive compatibility constraint of participating in the competition can be obtained.


(7) $$\frac{\partial EU}{\partial e}=f\left(0\right)\left(U_{H}-U_{L}\right)$$

Among


(8) $$U_{iH}=\left(W_{H}-c\left(e_{i}\right)\right)-\beta \left(W_{H}-c\left(e_{i}\right)\right)$$

(9) $$U_{iL}=\left(W_{L}-c\left(e_{i}\right)\right)-\alpha \left(W_{H}-c\left(e_{i}\right)\right)$$

By substituting the balanced effort level, we get


(10) $$\frac{\partial U_{iH}}{\partial e_{i}}=\left(1+\beta \right)c$$

(11) $$\frac{\partial U_{iL}}{\partial e_{i}}=\left(1-\alpha \right)c$$

In equilibrium, the utility of the winner and the loser is


(12) $$U_{H}=\left[W_{H}-c\right]-\beta$$

(13) $$U_{L}=\left[W_{L}-c\right]-\alpha$$

Get the functional relationship between the salary gap and the participants’ balanced effort


(14) $$f\left(0\right)+\frac{1}{2}\left(\beta -\alpha -2\right)=0$$

(15) $$\Delta W=\frac{\left(2+\alpha -\beta \right)}{2f\left(0\right)}$$

There is a functional relationship between the agent’s equilibrium effort level and the pay gap. In particular, when the existence of fairness preference is not considered, α=β=0, the functional relationship is simplified to f(0)⋅ΔW=c.

The only way for social work to better serve employees and enterprises is to truly integrate into the enterprise. The only effective way to maximize the role of enterprise social work is to effectively integrate social work into enterprise management. It should be emphasized that the social work of enterprises is not exactly the same as the work of trade unions. Trade unions are the main body of trade union work organizations. Trade unions represent the broad working class and belong to the willing behavior of the working class. Their purpose is to safeguard the rights and interests of workers. Relying on the form of collective, negotiating or negotiating with enterprises with equal characteristics is a way for trade unions to help workers.

## An economic study on the causes of employees’ psychological stress

### Data analysis

This paper analyzes two kinds of personnel. First, managers. They generally have higher personal income. In the face of information uncertainty, their personal income is higher than that of ordinary people. For them, increasing their income by the same amount will not give them much incentive. Utility increases with the increase of income. Utility decreases marginally with the increase of income. Second, ordinary employees. Their income is relatively low. Under other similar conditions, low basic income makes them pay more attention to income. That is, the diminishing speed of marginal utility is not as fast as that of managers, which is reflected in the radian of the utility curve.

For the convenience of analysis, it is assumed that all people make the same type of decision. They have the same degree of grasp of their own decision (that is, they also know the uncertainty and risk distribution of making the decision). The difference is that the benefits of the decision are different and the resulting income utility is different. All survey data sources are from Internet databases. All employees’ learning performance measurement items are above the standard value; At the same time, it can be seen from the value that deleting any item in the communication effectiveness measurement scale will not significantly improve the overall performance of the scale; Finally, the value of the overall employee learning performance measurement scale is, indicating that the reliability of the scale is good. The results of analysis and reliability test of employees’ learning performance are shown in Table 1.

**Table 1:** Analysis and reliability test of employees’ learning performance.

Item	Corrected item-total correlation	Alpha if tem deleted	Alpha
ELP1	0.621	0.826	0.848
ELP2	0.581	0.832	
ELP3	0.616	0.826	
ELP4	0.588	0.831	

The reliability of the measurement scale of the main variables, perceived differences, communication effectiveness, attitude towards diversity, individual innovative behavior, and employee learning performance are all higher than the level, that is, the reliability of the sample investigated in this paper is good. The reliability analysis results of the main scales used in this paper are shown in Table 2. The analysis and reliability test results of individual innovative behavior are shown in Table 3.

**Table 2:** Reliability analysis results of measurement scale.

Variable	Measurement item	Alpha
Attitude towards diversity	11	0.92
Employee learning performance	8	0.83
Perception difference	9	0.81
Communication effectiveness	14	0.93

**Table 3:** Analysis and reliability test of individual innovative behavior.

Item	Corrected item-total correlation	Alpha if tem deleted	Alpha
EIB1	0.825	0.911	0.928
EIB2	0.792	0.916	
EIB3	0.764	0.921	
EIB4	0.781	0.917	

The values of all individual innovation behavior measurement items are above 0.7, exceeding the standard value of 0.30; At the same time, it can be seen from the value that deleting any item in the communication effectiveness measurement scale will not significantly improve the overall performance of the scale; Finally, the value of the overall scale of individual innovation behavior is 0.9024, which indicates that the reliability of the scale is good.

**Figure 3:**
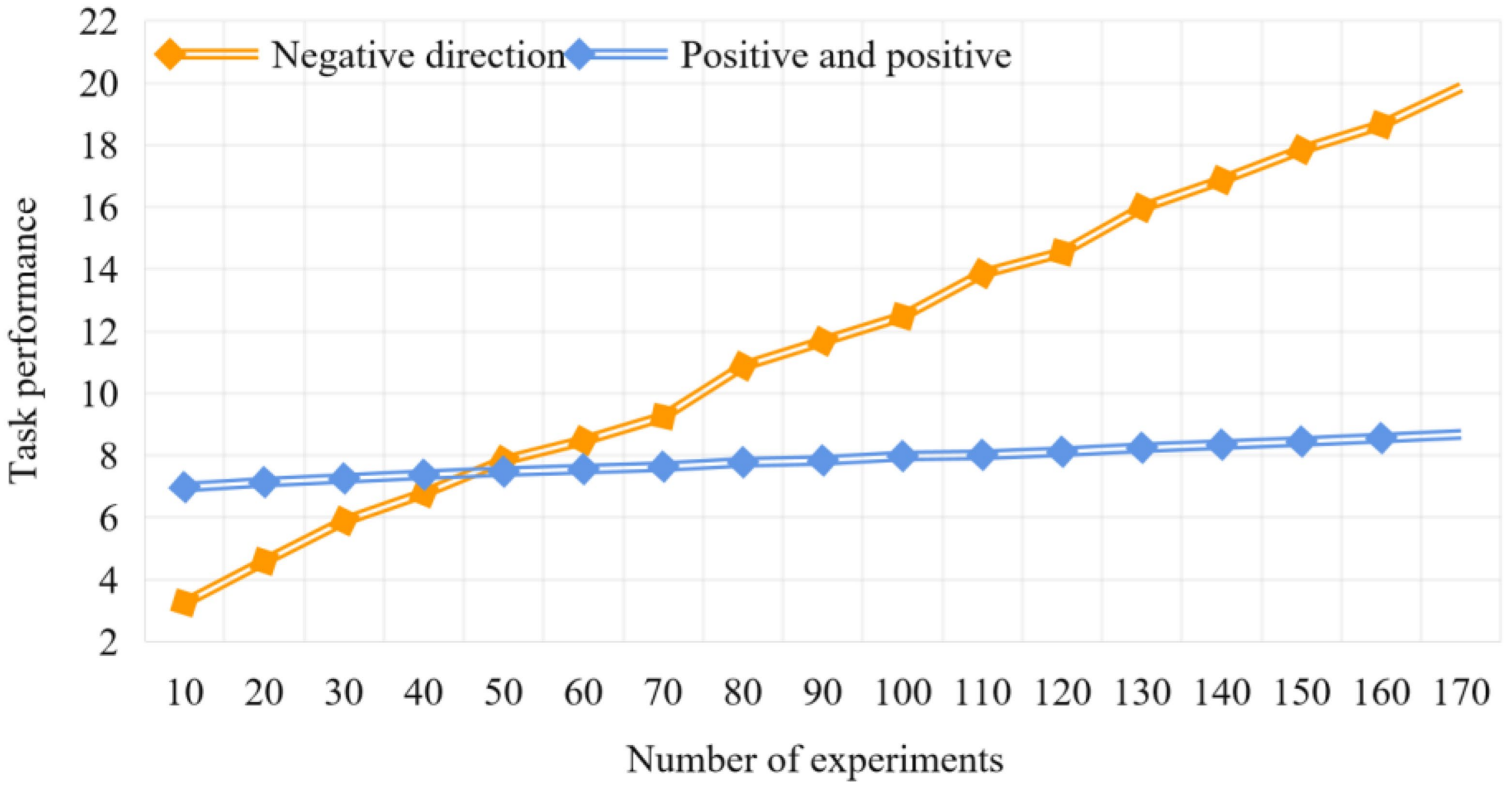
Adjustment of employee and team task performance in enterprises with heterogeneous attitudes.

The process of ANOVA includes the establishment of original hypothesis and bezawa hypothesis. The original assumption is that the corresponding mean values of different levels are equal. Then give that significant level *α*, the default is 0.05. Calculate *F*0 value of *F* statistic (*f*0 = error degree of freedom/model degree of freedom). The assumption condition of variance is that the residual obeys normal distribution, and its condition is equivalent to: (1) each group of observations obeys normal distribution (if the number of observations is enough, it is considered as normal distribution). (2) Homogeneity of variance. (3) The observations in the data are independent. The related hypotheses were tested by variance analysis of the impact of team heterogeneity structure and team members’ attitude towards diversity on team task performance. Before the variance analysis test, this paper carried out the variance homogeneity test. In order to reveal the essence of the adjustment effect, we drew a simple effect analysis chart according to the degree of team heterogeneity and the mean value of the attitude towards heterogeneity. Building a modern employee performance appraisal system is the foundation. Building an employee performance appraisal system that meets the requirements of modern enterprise system is the foundation. To build this system, we should grasp the following points, and enterprise leaders should give full support. All employees should actively participate. Performance appraisal must be combined with the development strategy of the enterprise. The assessment standards must be clear, clear and measurable. Set according to the length of the performance reflection cycle. (1) For enterprises that implement target management, it can be 1 year or more, or half a year, quarterly or monthly. (2) For enterprises that implement the contract system, the whole contract period can be regarded as the evaluation cycle, and the contract period can also be divided into several stages as the evaluation interval. (3) For enterprises that implement the contract system, the whole contract period can be used as the evaluation cycle, and the contract period can also be divided into several stages as the evaluation interval, which can be set according to the purpose of the evaluation. The adjustment of the task performance of employees and teams with the attitude towards heterogeneity is shown in Figure 3.

As can be seen from Figure 3, for those teams with positive attitude towards heterogeneity, the task performance gap between those teams with overlapping heterogeneity attributes and those with cross heterogeneity attributes is not obvious; However, for those teams with negative attitude towards heterogeneity, the task performance gap between those with overlapping heterogeneity attributes and those with cross heterogeneity attributes is very obvious.

**Figure 4:**
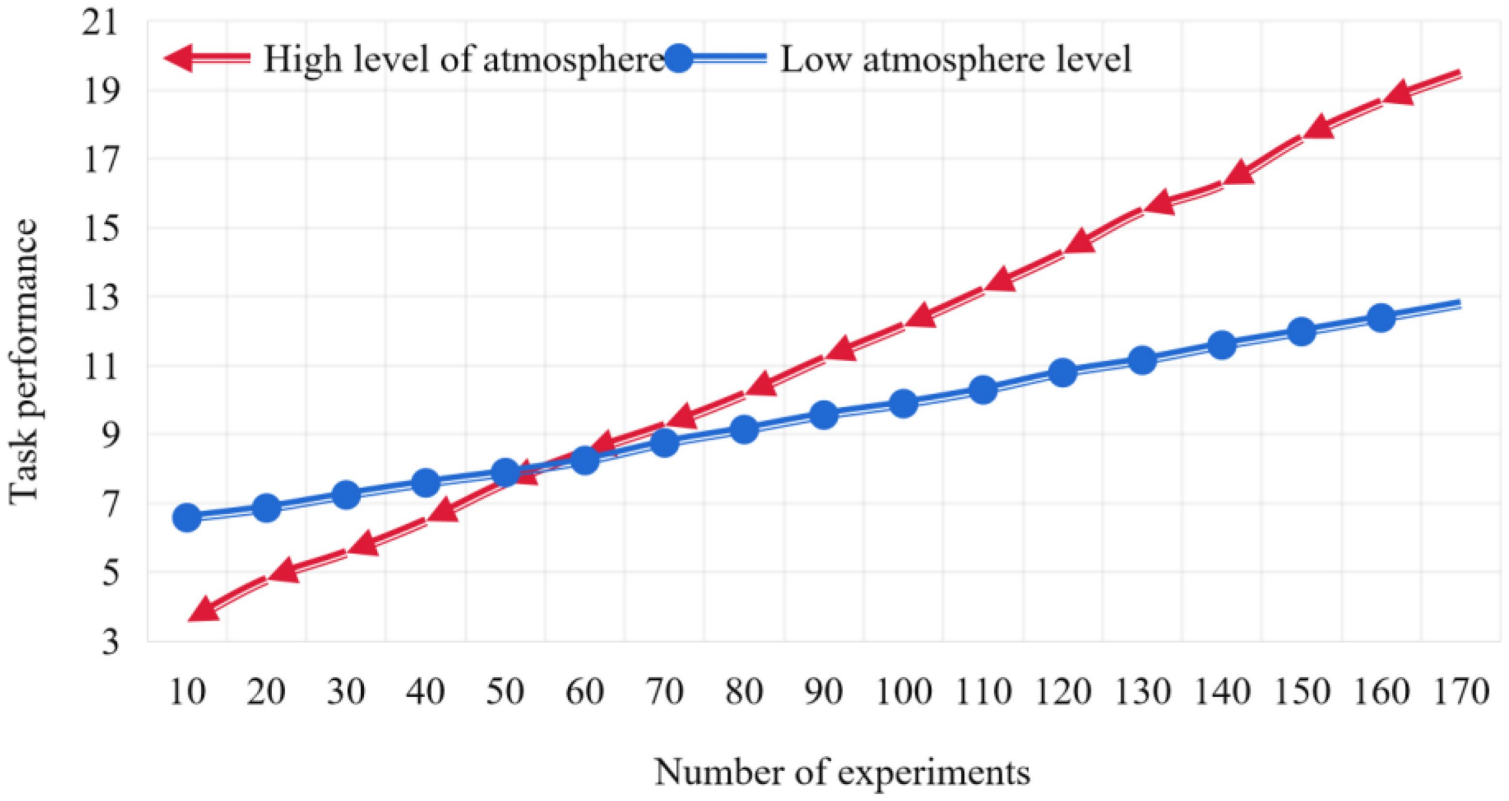
Adjustment of team open enterprise employee and team task performance.

As for the regulating effect of team openness climate on team heterogeneity and team task performance, since the degree of team heterogeneity is a classified variable, and the data of team openness climate collected through the scale is a continuous variable, we conduct regression analysis on the result variables by setting dummy variables on the degree of heterogeneity, so as to test the regulating effect of team openness climate. In order to reveal the essence of the adjustment effect, we draw a simple effect analysis chart according to the value of the regression equation. The adjustment of team open enterprise staff and team task performance is shown in Figure 4.

As can be seen from Figure 4, for those teams with a high level of team openness, with the improvement of team heterogeneity, team task performance has an obvious upward trend; For those teams with low level of team openness, with the improvement of team heterogeneity, team task performance shows a slight upward trend. It can be clearly seen from the experiment that when the number of experiments reaches 100, the task performance of high atmosphere level is 12.14, and that of low atmosphere level is only 9.89. Therefore, the atmosphere of team employees is very important for team task performance.

### Simulation and result analysis

This paper adopts the clustering method of emotional characteristics of mental health, and the distance measurement uses cosine similarity, with the number of categories *k* = 6, in order to compare with the predefined categories. Because emotional feature clustering is largely affected by the selection of initial points. This paper selects 15 groups of initial center points for experiments, and finally takes the average value of 15 clusters. Since the number of topics represented by emotional features will affect the clustering results, in order to obtain better clustering results, the best number of topics is selected through experiments, as shown in Table 4.

**Table 4:** Cluster purity and F value expressed by emotional features.

Number of topics	40	50	60	70
Purity	0.6312	0.6486	0.6524	0.6784
*F* value	0.6214	0.6312	0.6402	0.6747
Number of topics	80	100	110	120
Purity	0.6873	0.6796	0.6724	0.6605
F value	0.6730	0.6691	0.6682	0.6596

As can be seen from Table 4 that the clustering effect is better when the number of topics is 70. Therefore, the experimental results with the number of topics of 70 are taken as the clustering results of emotional characteristics. Using the above emotional feature clustering method, the causes of psychological stress of employees in the industry are clustered. The average value of 20 clustering experiments is shown in Table 5.

**Table 5:** Purity and F value of clusters under different characteristic representations.

Experiment purity	Boolean representation	Clustering representation of affective features	Expression of characteristic emotion tendency		
			Only 0	O + SO	O + SO(^*^)
*F* value	0.6546	0.6873	0.7052	0.7934	0.8122
Experiment	0.6214	0.6730	0.6846	0.7545	0.7805

As can be seen from Table 5 that the purity and *F*-value of clustering are obviously improved after adding sentence tendency to different expressions of characteristic emotion tendency, which indicates that this method of characteristic expression is effective. Effective combination of features can appropriately reduce the redundancy among features, and to some extent, reduce the sparseness of text feature matrix caused by too many features, and improve the purity and *F* value of clustering.

**Figure 5:**
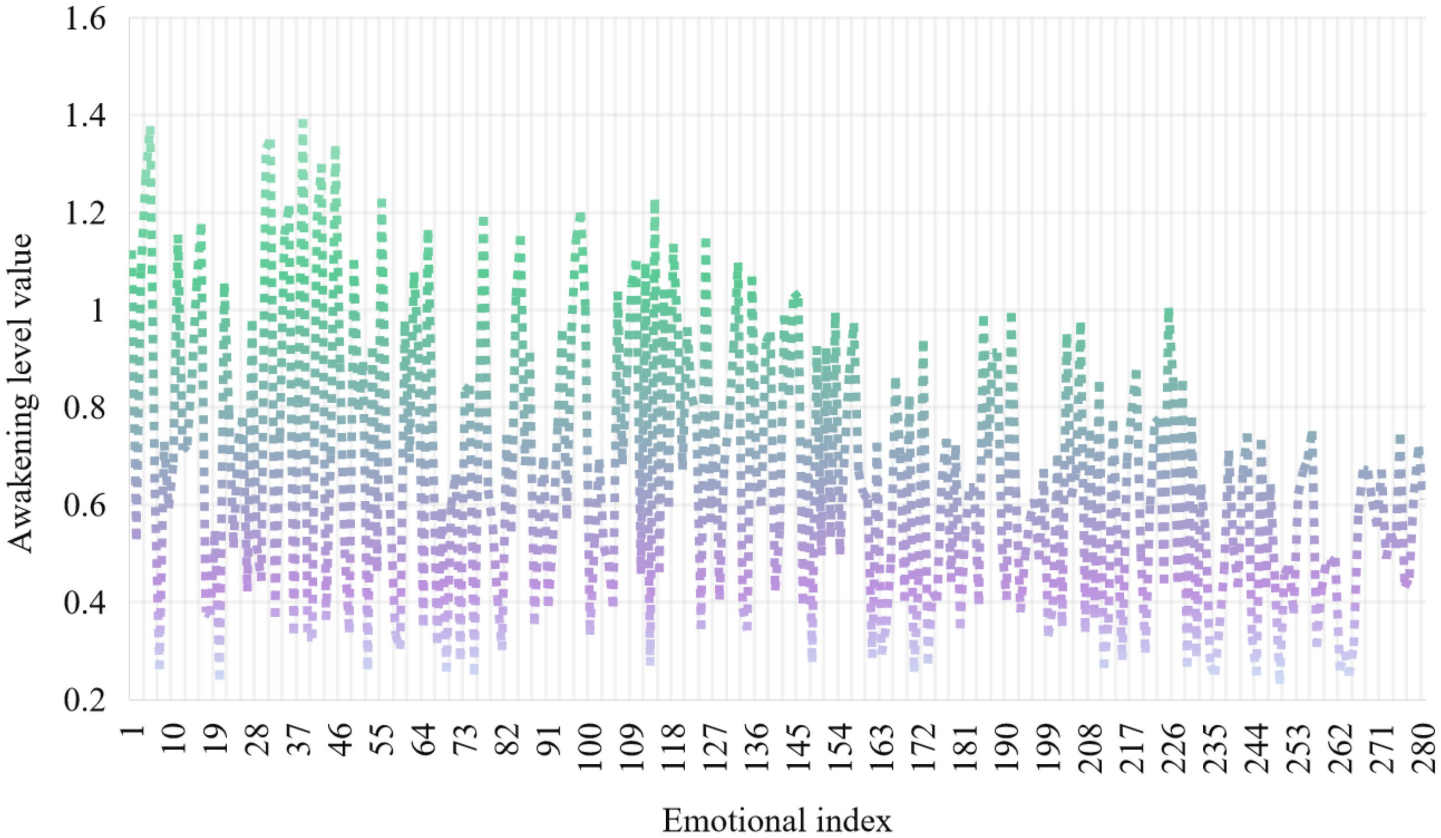
Distribution of emotional intensity under the control dimension.

According to the characteristics of emotion analysis, this experiment analyzes the causes of employees’ psychological stress. Controlled emotion is composed of emotional structure dimension and arousal level, that is, intensity. In order to observe the characteristics of controlled emotion more intuitively, the intensity of each emotion is controlled after the emotional types in the dimension are sorted by frequency weight from big to small. An experimental test was conducted on the emotional intensity under the control dimension, and the experimental results are shown in Figure 5.

As can be seen from Figure 5, the arousal level of emotion is not very high in the control dimension, and gradually decreases with the increase of emotion index. In the environment where the three basic psychological needs are not met, people mainly feel that the types of negative emotions and arousal level are not high.

**Figure 6:**
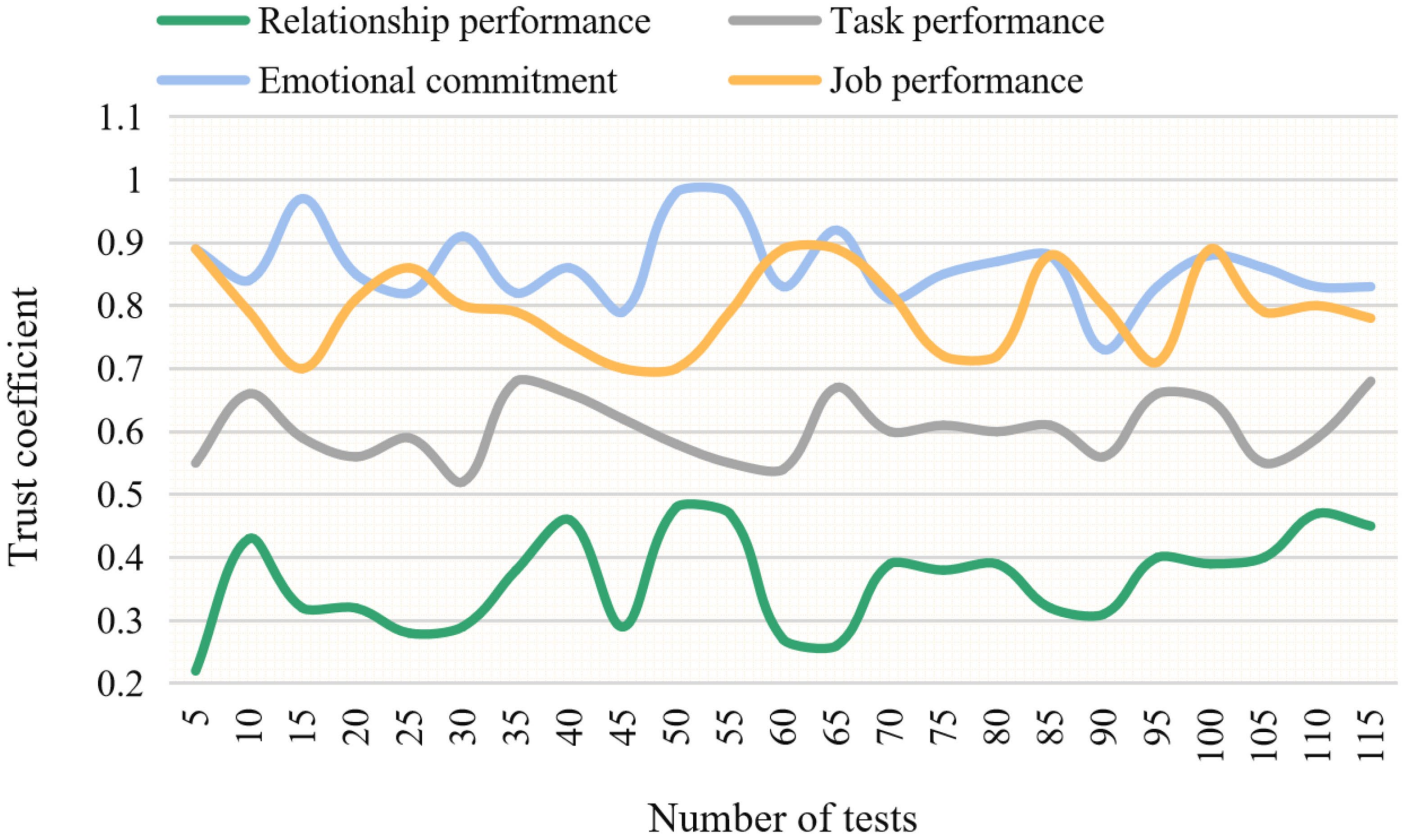
Variation of reliability coefficient under different items.

**Figure 7:**
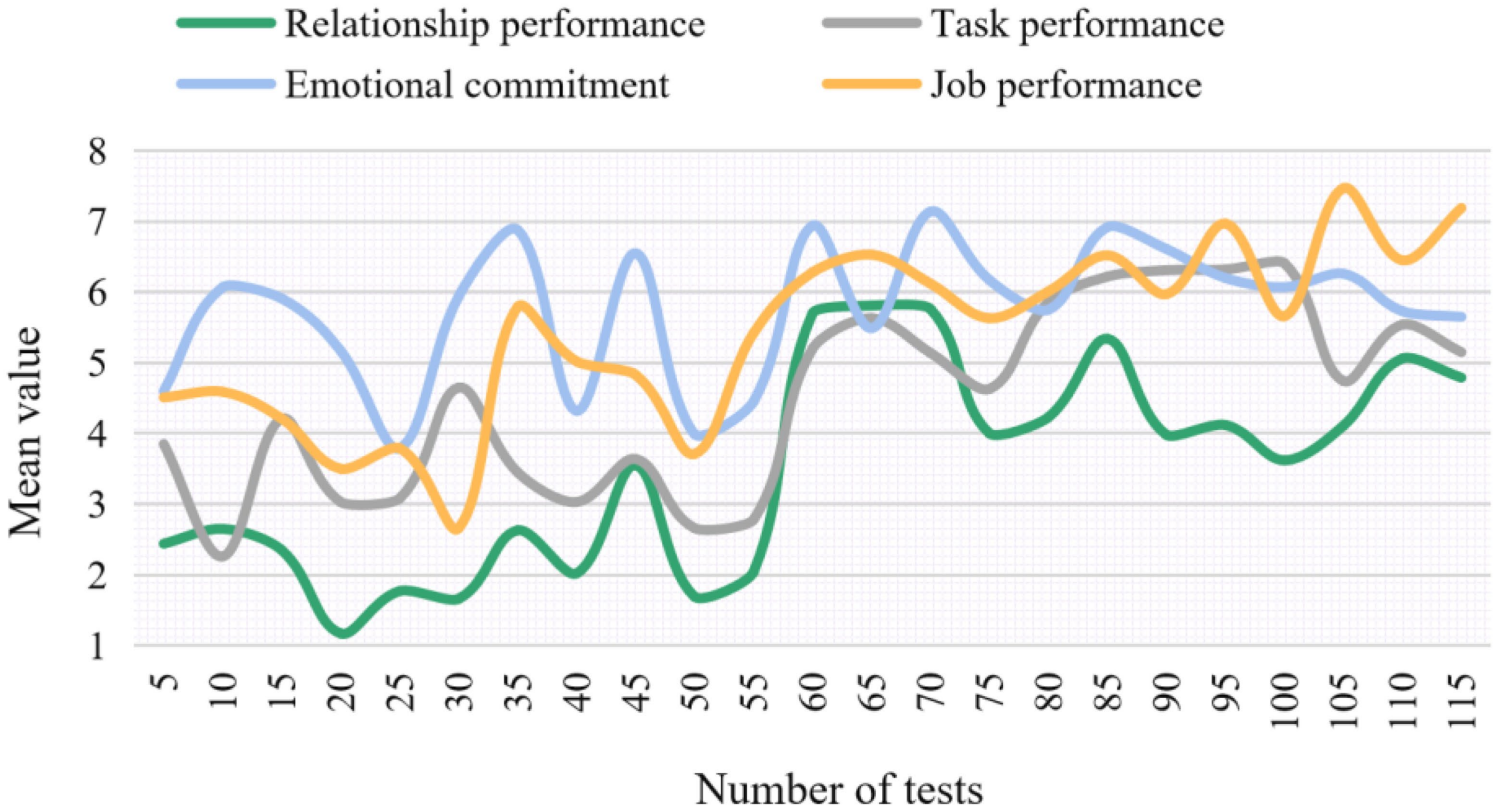
Mean change under different items.

Reliability is an important index to measure the quality of the questionnaire, which is used to measure the reliability and stability of the questionnaire. In this paper, the five-level scale is used to measure the variables, and the following four items are adopted to carry out the experiment: relationship performance, task performance, emotional commitment and job performance, and the mean values are also compared. The experimental results of the change of reliability coefficient under different items are shown in Figure 6. The change of the mean value under different items is shown in Figure 7.

As can be seen from Figure 6, when the number of experiments reaches 50, the reliability coefficient values are relationship performance 0.42, task performance 0.66, work performance 0.84 and emotional commitment 0.94, respectively. All the reliability coefficients of the questionnaire are close. All the items of the questionnaire passed the reliability test.

As can be seen from Figure 7, the average of emotional commitment is about 5.76, the average of relational performance is about 3.50, the average of task performance is about 4.51, and the average of job performance is about 5.43. It can be seen that the employees’ emotional commitment and job performance level of this survey object are relatively high. It shows that the data results of the research object are relatively stable and concentrated. On the other hand, it shows once again that the method proposed in this paper has certain stability.

This paper analyzes the emotional feature vectors of 150 employees’ psychological stress. Firstly, it makes correlation analysis. We use Pearson correlation coefficient to calculate the correlation among variables. Firstly, the data of each column is standardized, that is, the data of each column is subtracted from the average value of the column and divided by the standard deviation. The formula is as follows:


(16) X=(x−e(x))/sd

Then the covariance formula is used to calculate the correlation coefficient.


(17) R(x,y)=cor(x,y)=x.y

The correlation coefficient matrix of each feature is shown in Table 6.

**Table 6:** Correlation coefficient matrix of each feature.

1	−0.23	0.43	−0.55
−0.23	1	−0.27	0.48
0.43	−0.27	1	−0.15
−0.55	−0.48	−0.15	1

As can be seen from Table 6 that the first column of the user’s emotional feature vector is divided by the third column and the second column by the fourth column. We name the first column of the new feature vector positive emotional direct coefficient and the second column negative emotional direct coefficient. The emotional direct coefficient reflects the directness of the user’s speech, and the higher it is, the more direct the user speaks.

**Figure 8:**
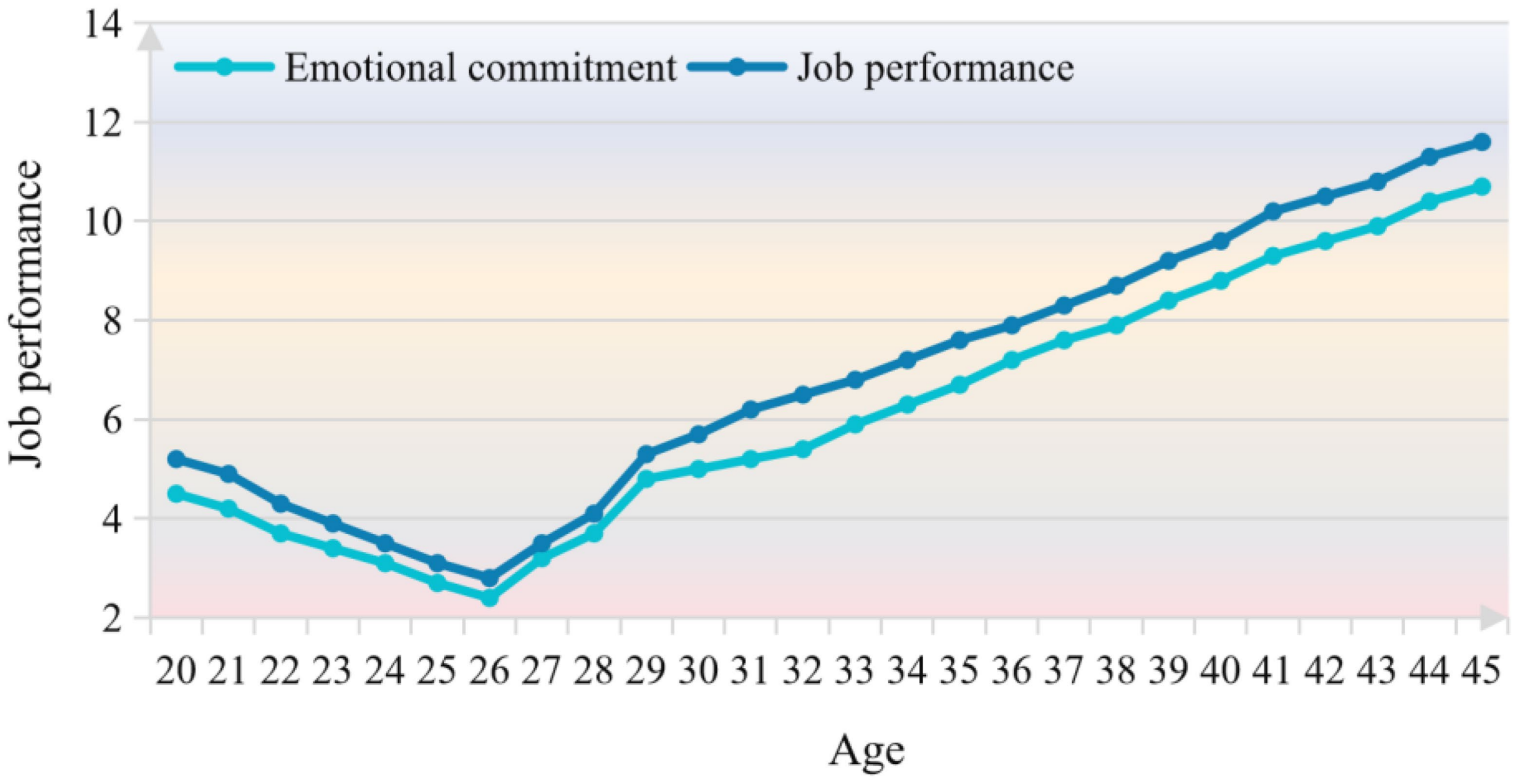
Changes of employees’ emotional commitment and job performance level with age.

The emotional commitment and job performance of employees of different ages, as well as their two dimensions, organizational emotional commitment and supervisor emotional commitment, have certain trends, with age as the horizontal axis and employee commitment level as the vertical axis. There is a general pattern between the two variables, and the lowest point is the commitment level of employees in the age group of “over, under.” That is, the level of employees’ emotional commitment, organizational emotional commitment and supervisor’s emotional commitment in the age group of “over, under” is lower than the overall commitment level. The changing trend of employees’ job performance and its dimensions at different ages is similar to the trend of emotional commitment. The changes of employees’ emotional commitment level and job performance level with age are shown in Figure 8.

As can be seen from Figure 8, with the increase of age, employees with high emotional commitment to the organization and supervisor are more likely to stay in the enterprise. Among them, the commitment level of employees under the age of 26 is not at the lowest point, probably because among the subjects in this paper, the employees in this age group have relatively low academic qualifications, relatively simple ideas, and pay more attention to their current jobs. Compared with college graduates who have just joined the work, they are more likely to reach an agreement with the organization emotionally. As can be seen from the figure, the changing trend of employees’ job performance and its dimensions at different ages is very similar to the trend of emotional commitment. However, it is worth noting that the distribution of the number of subjects in different age stages of the subjects in this paper is basically a pattern, so the differences in emotional commitment and job performance caused by age may be related to the age structure characteristics of the subjects in this paper.

## Conclusion

In modern society, in enterprises, the psychological health pressure of enterprise employees is inevitable. According to the inverse theory of work pressure and work performance, too high or too low work pressure will reduce efficiency and have a negative impact on work performance. Therefore, based on the clustering of emotional characteristics, this paper analyzes the causes of employees’ psychological pressure from the perspective of economics. For teams with low team openness, team task performance shows a slight upward trend with the improvement of team heterogeneity. It is obvious from the experiment that when the number of experiments reaches 100, the task performance of high atmospheric level is 12.14, and that of low atmospheric level is only 9.89. Therefore, for team task performance, the atmosphere of team employees is very important. Therefore, enterprises should regularly evaluate employees’ stressors and stress tolerance, and appropriately increase or reduce excessive stress according to employees’ work stress level and psychological tolerance, so as to maximize employees’ performance. After clustering analysis of employees’ mental health characteristics, it not only increases employees’ spare time life, but also reduces employees’ daily contradictions, alleviates employees’ work pressure, and becomes a platform for employees to improve understanding and a promoting factor for harmonious employee relations. However, in the process of practice, there are still some insurmountable difficulties. Some employees did not cooperate, because the survey indicators designed the privacy of employees. Therefore, the data is still flawed.

## Data availability statement

The raw data supporting the conclusions of this article will be made available by the authors, without undue reservation.

## Author contributions

BL: conceptualization, formal analysis, and writing-original draft. YQ: methodology, data curation, and writing-original draft. All authors contributed to the article and approved the submitted version.

## Conflict of interest

The authors declare that the research was conducted in the absence of any commercial or financial relationships that could be construed as a potential conflict of interest.

## Publisher’s note

All claims expressed in this article are solely those of the authors and do not necessarily represent those of their affiliated organizations, or those of the publisher, the editors and the reviewers. Any product that may be evaluated in this article, or claim that may be made by its manufacturer, is not guaranteed or endorsed by the publisher.
